# DDBJ Read Annotation Pipeline: A Cloud Computing-Based Pipeline for High-Throughput Analysis of Next-Generation Sequencing Data

**DOI:** 10.1093/dnares/dst017

**Published:** 2013-05-08

**Authors:** Hideki Nagasaki, Takako Mochizuki, Yuichi Kodama, Satoshi Saruhashi, Shota Morizaki, Hideaki Sugawara, Hajime Ohyanagi, Nori Kurata, Kousaku Okubo, Toshihisa Takagi, Eli Kaminuma, Yasukazu Nakamura

**Affiliations:** 1 Center for Information Biology and DNA Data Bank of Japan, National Institute of Genetics, 1111 Yata, Mishima, Shizuoka 411-8510, Japan; 2 Fujisoft Incorporated, 3 Kanda-neribeicho, Chiyoda-ku, Tokyo 101-0022, Japan; 3 Plant Genetics Laboratory, National Institute of Genetics, 1111 Yata, Mishima, Shizuoka 411-8510, Japan; 4 Database Center for Life Science, 2-11-16 Yayoi, Bunkyo, Tokyo 113-0032, Japan

**Keywords:** next-generation sequencing, sequence read archive, cloud computing, analytical pipeline, genome analysis

## Abstract

High-performance next-generation sequencing (NGS) technologies are advancing genomics and molecular biological research. However, the immense amount of sequence data requires computational skills and suitable hardware resources that are a challenge to molecular biologists. The DNA Data Bank of Japan (DDBJ) of the National Institute of Genetics (NIG) has initiated a cloud computing-based analytical pipeline, the DDBJ Read Annotation Pipeline (DDBJ Pipeline), for a high-throughput annotation of NGS reads. The DDBJ Pipeline offers a user-friendly graphical web interface and processes massive NGS datasets using decentralized processing by NIG supercomputers currently free of charge. The proposed pipeline consists of two analysis components: basic analysis for reference genome mapping and *de novo* assembly and subsequent high-level analysis of structural and functional annotations. Users may smoothly switch between the two components in the pipeline, facilitating web-based operations on a supercomputer for high-throughput data analysis. Moreover, public NGS reads of the DDBJ Sequence Read Archive located on the same supercomputer can be imported into the pipeline through the input of only an accession number. This proposed pipeline will facilitate research by utilizing unified analytical workflows applied to the NGS data. The DDBJ Pipeline is accessible at http://p.ddbj.nig.ac.jp/.

## 1. Introduction

Next-generation sequencing (NGS) is an increasingly important technology in genome and molecular biology research, partly because of its rapidity, precision, and cost effectiveness.^1–4^ NGS technology allows several analyses such as resequencing, *de novo* assembly of genomes, transcriptome analysis, Chromatin Immunoprecipitation (ChIP) sequencing, and exome analysis.^5^ With ever-decreasing sequencing costs, NGS read datasets can now reach terabase sizes. These massive sequencing datasets demand high-performance computational resources, rapid data transfer, large-scale data storage, and competent data analysts. This increase in scale appears to impede data mining and analysis by researchers.

The DDBJ Sequence Read Archive (DRA), released in 2009, is a data archive for NGS raw reads that has been maintained at the DNA Data Bank of Japan (DDBJ) of the National Institute of Genetics (NIG).^6,7^ The DRA is a global provider of public nucleotide sequences in partnership with the International Nucleotide Sequence Database Collaboration (INSDC)^8^ consisting of the Sequence Read Archive (SRA) of the National Center for Biotechnology Information (NCBI) in the USA^9^ and the European Read Archive (ERA) of the European Bioinformatics Institute (EBI) in Europe.^10^ Researchers may wish to reuse massive read datasets in DRA; however, their DRA file size tends to be too large to be downloaded to a local computer.

A computational system known as the ‘cloud’, consisting of data service provided via the Internet, was recently developed. Cloud computing allows users to avail services provided by data centres without building their own infrastructure. The infrastructure of the data centre is shared by a large number of users, reducing the cost to each user. To manage the flood of NGS data, several large-scale computing platforms have been recommended.^11–13^ Cluster computing is performed by multiple computers typically linked through a fast local area network and functioning effectively as a single computer. Grid computing is performed by loosely coupled networked computers from different administrative centres that work together on common computing tasks. Cloud computing is the computing ability that abstracts away the underlying hardware architecture and enables convenient on-demand network access to a shared pool of computing resources that can be readily provisioned and released. In particular, a model of cloud computing, Software as a service (SaaS), is referred to as ‘on-demand software’ and is available via a web browser. Cloud computing is a system uniting clusters of personal computers linked together similar to grid computing. The hallmark of cloud computing is that the users can perform computation across the Internet, without the necessity of understanding the underlying architecture.

The DDBJ Read Annotation Pipeline (DDBJ Pipeline) was released in 2009 with the aim of supporting users wishing to submit NGS data analysis results to the DDBJ database, a cloud computing-based analysis pipeline for DRA NGS data. This pipeline comprises two analytical components: a basic analytical process of reference mapping and *de novo* assembly and a process of multiple high-level analytical workflows. The main workflows of the high-level analysis offer structural and functional annotations. The DDBJ Pipeline, which is a web application based on the SaaS model of cloud computing, assists in the submission of analysed results to DDBJ databases by automatically formatting data files and facilitates the web-based operation of NIG supercomputers for high-throughput data analysis. Although conventional web-based genome-analysis pipelines, such as NCBI Prokaryotic Genomes Automatic Annotation Pipeline (PGAAP)^14^ and Rice Genome Automated Annotation System (RiceGAAS),^15^ perform genomic annotation of a draft sequence, their main target is Sanger-based sequence reads in small datasets. In contrast, the DDBJ Pipeline processes multiple datasets of terabase size using the computational resources of NIG supercomputers (the system is introduced in http://sc.ddbj.nig.ac.jp/index.php/en/).

In this report, we introduce the DDBJ Pipeline system with respect to its hardware and software configuration and outline its usage statistics since 2009. At present, the NIG supercomputer time is provided free of charge. We believe that provision of computational services for NGS data analysis without cost to users will increase the use of public data and accelerate data submission to public databases.

## 2. Materials and methods

### 2.1. Basic analysis

The pipeline accepts single- or paired-end reads in FASTQ^16^ format and simple metadata describing the organism and experimental conditions associated with the reads. The type of sequencer is immaterial, providing that the data format of the reads is followed. Users submit their NGS data and XML-formatted metadata to the DRA. They may also submit the NGS data to a DDBJ Pipeline directory. Users subsequently analyse the NGS data in the pipeline using the accession numbers. The FASTQ-formatted sequences and metadata are loaded from the DRA databases. The DDBJ Pipeline allows pre-processing by trimming low-quality bases from both ends of the reads. The pre-processing function returns statistics and figures describing read qualities by read position, and these outputs enable users to set trimming parameters. The FASTQ files are used either for genome mapping or for *de novo* assembly. The basic analysis supports various mapping and *de novo* assembly tools for the NGS data according to the user's preference. (Analytical programme tools hosted in the DDBJ Pipeline^17–36^ are listed in Table 1). Optional analytical parameters can be selected. Sequential commands from pre-processing to outputting analytical results are preset for easy operation. Reference data, such as the relevant genome sequence, can be retrieved from DDBJ databases by the Simple Object Access Protocol (SOAP).^37^ Users can confirm the error rate by read position and can trim low-quality bases from the reads. The numbers of mapped reads, genome coverage, depth, and maximum contig length are reported. Output files from all processing stages including SAM-formatted files,^24^ if supported by the tool, can be downloaded from an FTP server. A multiple FASTA file, which is convenient for subsequent submissions to the whole-genome shotgun (WGS) section of DDBJ, is built on the basis of consensus sequences from mapping or contig files from *de novo* assembly. The basic analysis system is built mainly using Perl 5.8, Java 6, PostgreSQL 8.3, and gnuplot (http://www.gnuplot.info/). Mapping and *de novo* assembly are performed on NIG supercomputers using 704 8-core 2.60-GHz Intel Sandy Bridge CPUs with 64 GB RAM and 1.6 TB storage, and 96 8-core 2.66-GHz Intel Xenon CPUs with 10 TB RAM, respectively.

**Table 1.** Analysis programmes hosted in the DDBJ Pipeline

Analysis type	Usage	Analysis tools
Basic analysis	Mapping	BLAT^17^
		BWA program^18,19^
		SOAP2^20^
		Bowtie^21^
		Bowtie 2^22^
		TopHat^23^
		SAMtools^24^
	*de novo* assembly	Velvet^25,26^
		SOAPdenovo^27^
		ABySS^28^
		Trinity^29^
High-level analysis	Web application	Galaxy^30^
	Annotation of mapping results	ANNOVAR^31^
		Cufflinks^32^
		MACS^33^
	Annotation of *de novo* assembled contigs	GENSCAN^34^
		GeneMark.hmm^35^
		BLAST^36^

Numbers in brackets following analysis tools are citations.

Mapping benchmarks in the basic analysis were calculated using the whole-genome NGS data from the Japanese rice cultivar ‘Omachi’ (paired-end reads of accession number DRR000719) and using the complete genome sequences of the Japanese rice cultivar ‘Nipponbare’ as reference (accession numbers NC_008394–NC_008405). *De novo* assembly was performed using whole-genome NGS reads of *Escherichia coli* NDM1Dok01 (paired-end reads of accession number DRR001003).

### 2.2. High-level analysis

Because advanced analysis after mapping and *de novo* assembly requires several workflows with variable functions, the high-level analysis system was mainly designed to use the Galaxy interface,^30^ a genomic workbench with a graphical user interface. To date, single-nucleotide polymorphism (SNP) analysis, transcriptome analysis (RNA-seq), and ChIP-sequencing have been implemented using SAMtools,^24^ Cufflinks,^32^ and MACS,^33^ respectively (Table 1). These analyses are performed using mapped results in the SAM format generated by the basic analysis. Users can modify parameter settings through the graphical user interface and execute the analysis flows repeatedly using Galaxy's Workflow and History methods. For SNP analysis, a figure showing the frequency distribution of SNPs over the entire genome can be produced. For transcriptome analysis, mapped results are sent to Cufflinks to quantify gene structures and expression values. In addition, these results can be visualized by genomic regions linked to the UCSC genome browser site (http://genome.ucsc.edu/).

The high-level analysis system requires the Galaxy environment, Cairo (http://www.cairographics.org/), and Perl modules from CPAN (http://www.cpan.org/) for graphical output. The analysis is performed on the same nodes as the mapping, using the 704 8-core 2.60-GHz Intel SandyBridge CPUs with 64 GB RAM and 1.6 TB storage.

## 3. Results and discussion

### 3.1. System configuration of the proposed pipeline

The system outline of the DDBJ Pipeline is summarized in Supplementary Fig. S1. Apache Tomcat and DB servers for the DDBJ Pipeline run on an NIG supercomputer, and an FTP server that handles data import and export and resides outside the supercomputer. Reads imported from the DRA are sent via its built-in FTP server.

The pipeline is built as a cloud computing-based web application, and its flow follows two steps. The basic analysis receives transferred reads and maps them to reference genomes or assembles them. The high-level analysis generates results closer to the research goals, such as genome contig construction, SNP detection, or expression analysis.

NGS data are transferred either to an analysis server for basic analysis or to Galaxy interface servers for high-level analysis, both residing within an NIG supercomputer. Classified on the basis of purpose, the data are analysed by the supercomputer nodes using the qsub command of the UNIVA grid engine.

### 3.2. A pipeline for high-throughput analysis of NGS data

**Figure 1.**
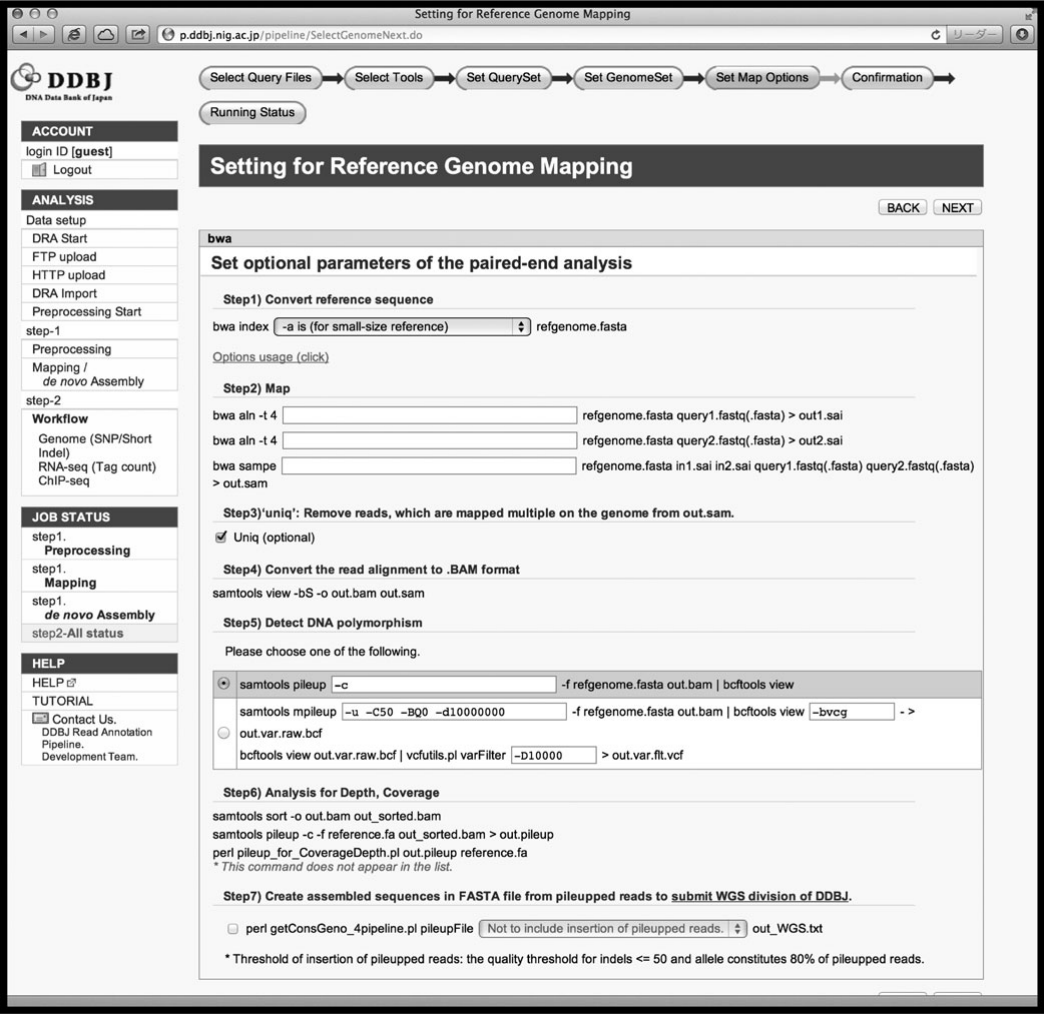
Interface for modifying the settings of analysis tools in basic analysis of the DDBJ Pipeline.

**Figure 2.**
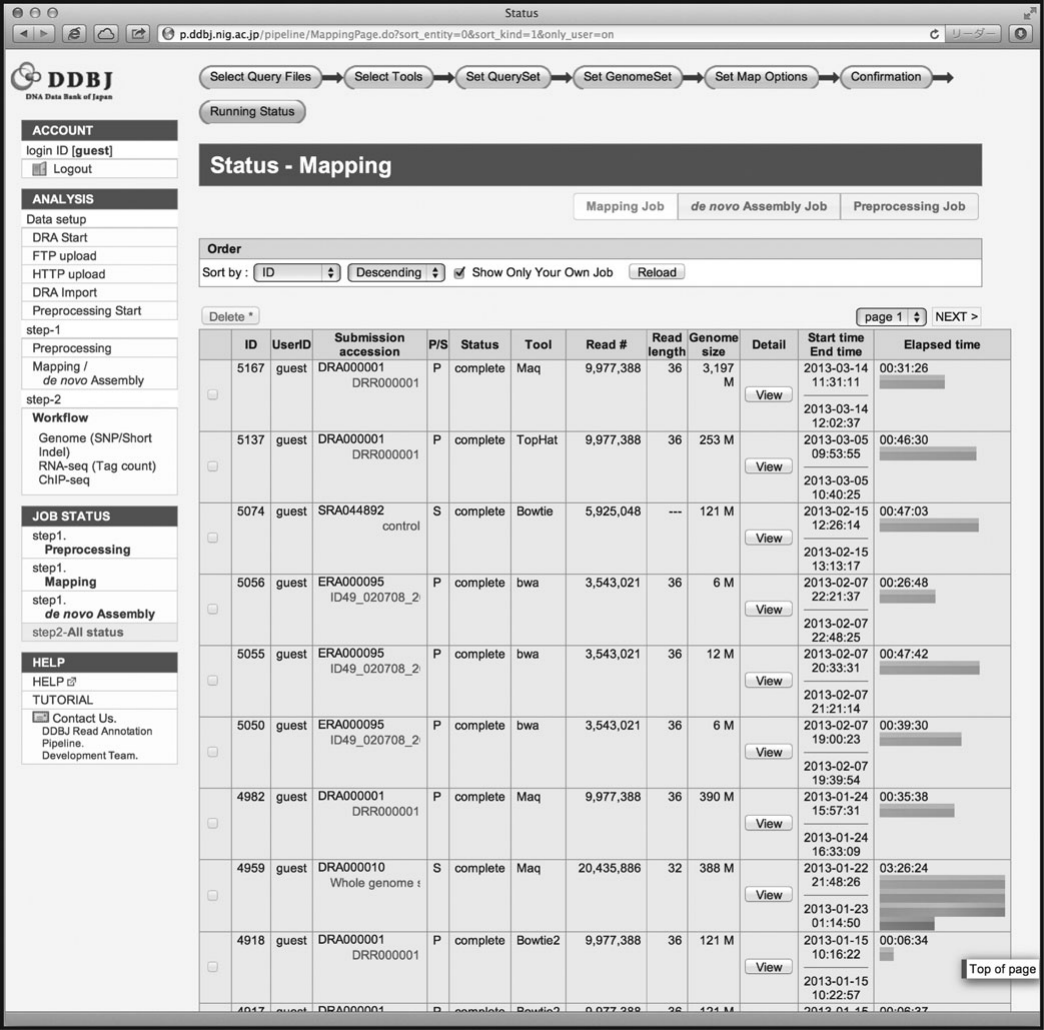
Job status list in basic analysis of the DDBJ Pipeline. Jobs executed in the DDBJ Pipeline are shown in lists, and users may manage the jobs, for example, by downloading results or by halting the jobs. The bars at the right end of the list indicate elapsed times.

In the basic analysis, the DDBJ Pipeline provides the following useful functions: (i) data transfer: at the start of analysis, users can specify three methods for query data: FTP uploading, secure copy from DRA if the data have been pre-registered to DRA, or HTTP uploading. If users wish to use public data as query data, they may choose directory upload from the DRA, whose data are shared with SRA and ERA. Public data may be used not only as query data, but also as reference sequences for mapping. (ii) Pre-processing in the form of trimming off low-quality parts of sequence reads: basecalling quality is not uniform and may influence mapping or assembly quality. Although trimming off less accurately identified bases is effective to maintain the quality, several analysis tools can be used as optional functions.^38–41^ The DDBJ Pipeline outputs trimmed reads and figures showing the distributions of read quality scores. (iii) Parameter changes to software components: the DDBJ Pipeline allows modification of some options and parameters of the software and allows users to limit reads to uniquely mapped reads in the SAM file by removing multiread sets (Fig. 1). (iv) Confirming job status and ensuring confidentiality: the DDBJ Pipeline communicates with web applications to analyses the NGS data using DDBJ supercomputers and currently supports 11 mapping or *de novo* assembly software packages (Table 1). During pre-processing, mapping, or *de novo* assembly on the supercomputer, users can confirm the status of their operation through a web browser (Fig. 2). The user's jobs are listed along with their status (‘running’, ‘complete’, ‘error’, etc.) and elapsed times. When the jobs are completed, the DDBJ Pipeline notifies users by e-mail. Output is not limited to SAM-formatted files as mapping results or FASTA files as assembly results, but includes intermediate files, work logs, and statistical data, including mapping coverage and depth or N50 contig size of assemblies. The DDBJ Pipeline and the NIG supercomputer system, which execute the pipeline jobs, may be accessed by an unspecified number of users. To protect user confidentiality, the DDBJ Pipeline does not allow users to identify any other user except for the demo user, and users may never access each other's queries except for public data and results.

As an example of a benchmark of the system, the DDBJ Pipeline enables the mapping of 34.7 million 75-base NGS reads to a 383-Mb reference genome using BWA program^18,19^ in 6.5 h and can assemble 24.4 million 80-base paired-end WGS reads in 10.5 min with SOAPdenovo,^27^ using the cloud computing system. NGS technologies reduce the cost and time required for sequencing, and the resulting data increase the submissions to public archives.^8^ Current developments in NGS technologies are leading to increases in both read length and the number of reads, and novel biological strategies are being developed to utilize the sequencing systems. The expansion of read numbers requires expanded computational resources, particularly for *de novo* assembly.^42^ The use of a computational cluster system allows decentralized processing, resulting in scalability for efficient analysis of NGS data. The DDBJ Pipeline supports not only the use of public domain data, but also the submission of mapping and assembly results to the WGS division of DDBJ in FASTA format. Basic analysis of the DDBJ Pipeline is accessible at http://p.ddbj.nig.ac.jp.

### 3.3. Genome-wide annotation by the high-level analysis of NGS data

The DDBJ Pipeline supports not only basic analyses, such as mapping, but also high-level analyses via the Galaxy interface, which has the advantage of modifiability and easy maintenance.^30^ User data such as login accounts, e-mail addresses, and passwords for access to Galaxy are shared with those of basic analysis. Therefore, basic analysis results, which are mapping or assembly jobs identified by job IDs, can be imported into Galaxy.

The high-level analysis has recently been augmented with the following four analysis methods:

- *SNP detection*: Users may view the pileup data produced by the basic analysis and identify SNPs using ANNOVAR.^31^ They may also view figures showing SNP distribution on the genome (Supplementary Fig. S2A and 2B).
- *RNA-Seq analysis*: Expression analysis using TopHat^23^ produces a SAM file that is subsequently processed using Cufflinks.^32^ Downloaded Cufflinks results are sent to the UCSC genome browser site (http://genome.ucsc.edu/cgi-bin/hgGateway),^43^ allowing the user to identify read expression patterns within genome-wide images. Cuffcompare, an analysis function of Cufflinks, is also available.
- *ChIP-Seq analysis*: The DDBJ Pipeline supports MACS^33^ for ChIP-Seq analysis.
- *Annotation of contigs by de novo assembly*: A length filter is applied to remove short fragments. For gene finding in contigs, gene prediction tools, such as GENSCAN for eukaryote and GeneMark.hmm for prokaryote data,^34,35^ are applied. BLASTX is used for similarity searching against known proteins^36^ (Supplementary Fig. S2C). Supplementary Figs S2A, S2B, and S2C showing the whole-genome distribution of SNPs or the functional annotation of assembled contigs provide researchers with inspiration for new discoveries. In addition, new strategies for applying NGS technologies to novel biological analyses will be developed in the future. Therefore, the high-level analysis has the flexibility to be modified.

The high-level analysis of the DDBJ Pipeline can be accessed at http://p-galaxy.ddbj.nig.ac.jp.

### 3.4. Usage statistics of the DDBJ Pipeline

As a beta version offering only the basic analysis component, the DDBJ Pipeline has been open to the public via the Internet with updates since August 2009. Some analytical tools have been replaced according to the frequency of their use since then. From the start of recording in June 2010, the number of jobs submitted was around 1800 (Table 2), not considering those used for development and demonstration. The number of mapping jobs (1428) was nearly quadruple that of *de novo* assembly jobs (326).

**Table 2.** Job numbers for the basic analysis of the DDBJ Pipeline (since June 2010)

Year	Pre-processing	Mapping	*de novo* Assembly	Total
2010	—^a^	674	35	709
2011	11	310	152	473
2012 (January–September)	33	444	139	616
Total	44	1428	326	1798

^a^Pre-processing was still under construction.

### 3.5. Building an environment supporting the use of NGS data by biologists

Deposits of NGS data in public databases (DRA, ERA, and SRA) are rapidly increasing each year.^6^ However, the NGS database has been used only as a data repository. Bioinformaticians use the data to test their own computer analysis programmes, whereas general biologists lacking computational skills rarely use the huge and unwieldy datasets. In this report, we present an example of how biologists may use public NGS data to their advantage. Genome sequences used as references for re-sequencing are often updated, resulting in the shifting of mapped positions of reads. Researchers may wish to compare SNPs that have been newly mapped and detected by them to previously detected SNPs in the public database. SNP positions in databases, such as dbSNP,^44^ based on older reference genomes will lead to confusion. The DDBJ Pipeline not only provides a computational environment for analysing NGS data, but also permits seamless access to the public domain data, including NGS short reads and complete genomes. Researchers familiar with the DDBJ Pipeline will be able to re-perform reference mapping quickly using public NGS reads with current genome sequences. It may occur that comparing or merging SNP data from their own dataset with the public data using the DDBJ Pipeline allows re-analysis with preferred parameters. We expect the DDBJ Pipeline to analyse users' NGS data, thereby accelerating the submission of NGS data to public databases such as DDBJ.

### 3.6. The cloud computational system for NGS data analysis

A recent cloud computing innovation is virtual machines (VMs), which are programmes that perform parallel processing to overcome differences between server platforms. VM technology has been used in bioinformatics.^11–13^ CloVR^45^ has been developed for analysing bacterial NGS data, and the RseqFlow workflow^46^ processes, RNA-Seq data. Cloud computing with VM expands genome informatics, and more tools will appear in future. Although the DDBJ Pipeline accommodates individual users' data, some biologists wish to host NGS analysis packages on their local servers to keep their data private. Therefore, we are studying the future incorporation of a VM package into the DDBJ Pipeline.

### Supplementary data

Supplementary Data are available at www.dnaresearch.oxfordjournals.org.

### Funding

This work was partially supported by a grant-in-aid for Scientific Research on Innovative Areas ‘Genome Science’ and Scientific Research (C) from the Ministry of Education, Culture, Sports, Science and Technology of Japan. The computations for this work were partially performed on the NIG supercomputer at ROIS National Institute of Genetics.

## Supplementary Material

Supplementary Data
