# Supplementary material for: DDBJ Read Annotation Pipeline: A Cloud Computing-Based Pipeline for High-Throughput Analysis of Next-Generation Sequencing Data
Source: DNA Res. 2013 May 8;20(4):383–90. doi: 10.1093/dnares/dst017 (PMC3738164; doi:10.1093/dnares/dst017)
Supplement: Supplementary Data [file supp_20_4_383__index.html]

DDBJ Read Annotation Pipeline: A Cloud Computing-Based Pipeline for High-Throughput Analysis of Next-Generation Sequencing Data — Supplementary Data 

# DDBJ Read Annotation Pipeline: A Cloud Computing-Based Pipeline for High-Throughput Analysis of Next-Generation Sequencing Data

## Supplementary Data

Supplementary Data

**Files in this Data Supplement:**

- Supplementary Data - Docx file
- Supplementary Figure 1 - eps file
- Supplementary Figure 2A - eps file
- Supplementary Figure 2B - eps file
- Supplementary Figure 2C - eps file
