# Supplementary material for: DDBJ Read Annotation Pipeline: A Cloud Computing-Based Pipeline for High-Throughput Analysis of Next-Generation Sequencing Data
Source: DNA Res. 2013 May 8;20(4):383–90. doi: 10.1093/dnares/dst017 (PMC3738164; doi:10.1093/dnares/dst017)
Supplement: Supplementary Data [file supp_dst017_dst017supp.docx]

**Supplementary Figure S1** Schema of the DDBJ Pipeline

The DDBJ Pipeline is composed of two parts: basic analysis, which includes mapping or *de novo* assembly of NGS data, and high-level analysis, which provides interpretations of the results of the basic analysis (e.g., SNP detection and expression analysis). High-level analysis is performed through Galaxy workflows [30]. The analyzed data are transferred by the DBI Connect module to the Galaxy interface. Numbers in brackets following analysis tools are citations.

**Supplementary Figure S2A** Image produced by high-level analysis of the DDBJ Pipeline (A)

SNP detection from pileup/mpileup formats of SAMtools [24].

**Supplementary Figure S2B** Image produced by high-level analysis of the DDBJ Pipeline (B)

Graphical representation of the chromosomal distribution of SNPs.

**Supplementary Figure S2C** Image produced by high-level analysis of the DDBJ Pipeline (C)

Output structural annotation of *de novo* assembly results. The two images represent results of gene prediction by GeneMark.hmm [35] on assembled contigs and similarity search by BLASTP [36] against predicted genes.
